# Data privacy protection in scientific publications: process implementation at a pharmaceutical company

**DOI:** 10.1186/s12910-022-00804-w

**Published:** 2022-06-25

**Authors:** Friedrich Maritsch, Ingeborg Cil, Colin McKinnon, Jesse Potash, Nicole Baumgartner, Valérie Philippon, Borislava G. Pavlova

**Affiliations:** 1 Baxalta Innovations GmbH, A Takeda Company, Vienna, Austria; 2 Takeda Pharmaceuticals International AG, Zurich, Switzerland; 3 Takeda Development Center Americas, Inc., Cambridge, MA USA

**Keywords:** Patient privacy, Reidentification risk, Clinical trials, Data de‑identification, Data sharing, Medical publications, Trial participant privacy

## Abstract

**Background:**

Sharing anonymized/de-identified clinical trial data and publishing research outcomes in scientific journals, or presenting them at conferences, is key to data-driven scientific exchange. However, when data from scientific publications are linked to other publicly available personal information, the risk of reidentification of trial participants increases, raising privacy concerns. Therefore, we defined a set of criteria allowing us to determine and minimize the risk of data reidentification. We also implemented a review process at Takeda for clinical publications prior to submission for publication in journals or presentation at medical conferences.

**Methods:**

Abstracts, manuscripts, posters, and oral presentations containing study participant information were reviewed and the potential impact on study participant privacy was assessed. Our focus was on direct (participant ID, initials) and indirect identifiers, such as sex, age or geographical indicators in rare disease studies or studies with small sample size treatment groups. Risk minimization was sought using a generalized presentation of identifier-relevant information and decision-making on data sharing for further research. Additional risk identification was performed based on study participant/personnel parameters present in materials destined for the public domain. The potential for participant/personnel identification was then calculated to facilitate presentation of meaningful but de-identified information.

**Results:**

The potential for reidentification was calculated using a risk ratio of the exposed versus available individuals, with a value above the threshold of 0.09 deemed an unacceptable level of reidentification risk. We found that in 13% of Takeda clinical trial publications reviewed, either individuals could potentially be reidentified (despite the use of anonymized data sets) or inappropriate data sharing plans could pose a data privacy risk to study participants. In 1/110 abstracts, 58/275 manuscripts, 5/87 posters and 3/58 presentations, changes were necessary due to data privacy concerns/rules. Despite the implementation of risk-minimization measures prior to release, direct and indirect identifiers were found in 11% and 34% of the analysed documents, respectively.

**Conclusions:**

Risk minimization using de-identification of clinical trial data presented in scientific publications and controlled data sharing conditions improved privacy protection for study participants. Our results also suggest that additional safeguards should be implemented to ensure that higher data privacy standards are met.

**Supplementary Information:**

The online version contains supplementary material available at 10.1186/s12910-022-00804-w.

## Introduction

Since their launch 20 years ago, publicly accessible clinical trial databases have proved to be a valuable resource of clinical trial information for participants, researchers, healthcare professionals and the general public. They have become an easily accessible and reliable source of information and have led to an increase in public trust in clinical trials [1–5]. In the past decade, the scientific and medical communities have emphasized the need for more rapid, easily accessible, and efficient dissemination of research outputs, leading funding agencies, publishers, and scientists to put forward open access principles [6–8]. Availability of these outputs via open access allows greater awareness of the latest data for healthcare practitioners and for researchers, patients, and patient advocates; it also encourages more rapid advances in research. However, the benefits of open access and data sharing must be weighed against the real risk of reidentification of individuals if data presented in scientific publications can be linked to information available in the public domain, therefore raising legitimate privacy concerns.

Several clinical trial data categories are publicly available. These include data derived from participant data and provided by research sponsors, academics or the pharmaceutical industry (e.g. data available from ClinicalTrials.gov, the European Union Drug Regulating Authorities Clinical Trials database [EudraCT], the European Network of Centres for Pharmacoepidemiology and Pharmacovigilance [ENCePP], European Union electronic Register of Post-Authorisation); data provided by regulatory authorities as part of transparency regulations (e.g. clinical study reports (CSRs), Module 2 clinical overviews/summaries in common technical document [CTD] submission packages); data provided via the use of medical devices during clinical trials; data provided by health authorities (e.g. public assessment reports); and data that were actively and knowingly provided by the study participants via social media, as well as in scientific publications that report treatment effects on study participants [9–11].

Current data protection laws and standards for anonymization consider that information on individuals covered in a data set must be protected for the data set to be considered anonymous [12, 13]. These regulations and laws, however, provide different data protection standards: applying either technology neutral approaches that allow the data controller or data processor to decide on the most reasonable method to use, based on the environment in which the data will be released [12, 14, 15], or technology specific anonymization methods, which provide fixed criteria in order for a dataset to be considered ‘de-identified’[13]. Data de‑identification is the process of anonymizing data sets before making them publicly accessible and has been the main method for sharing data while protecting people’s privacy [10]. The clinical trial data anonymization/de-identification standards that form part of data privacy regulations [10–12] are required because study participants could potentially be identified from sensitive parameters such as direct identifiers (e.g., participant identifiers, initials, study IDs), and indirect identifiers or quasi‑identifiers such as sex or gender, age, birth date, race, computer tomography scans, liver biopsy images (including time- or location-related information) [16, 17]. Descriptions of individual participants (e.g., demographic data for a single participant and highly characteristic data) within the context of the study and small group sizes can also increase a study participant’s risk of reidentification.

However, data anonymization and de-identification cannot fully prevent the reidentification of clinical trial participants. Studies have shown that reidentification of hospital discharge de‑identified data is possible using certain demographic parameters [18] and that participant ID code, year of birth, gender and ethnicity could uniquely identify the genomic data of study participants [19]. The fact that many supposedly anonymous data sets have been released and then reidentified has raised further concern about the confidentiality and privacy of participants in clinical trials. Personal data described in scientific publications may also be used to reidentify study participants (whether patients or volunteers), and the risk is highest for patients affected by a rare disease or condition [20].

Even when study participant data presented in scientific publications are anonymized and incomplete, there is still a substantial possibility that a specific individual could be correctly reidentified. A method that can accurately estimate the likelihood of a specific person being correctly reidentified, even in a thoroughly de-identified data set, has been reported [21]. Results obtained using this method have shown that reidentification is a real risk and that sampling or releasing partial data sets does not provide plausible deniability, that is, partial data sets alone are not sufficient to fulfil the duty to protect study participant privacy. This raises the question of whether existing de-identification practices and anonymization standards satisfy the urgent need for privacy in the current environment of increased transparency and data accessibility due to open access publishing [21].

Anonymized clinical trial research outputs available via open access and public information sources linked to study participant data presented in scientific publications would not automatically enable reidentification of study participants. However, the combination of such data from different sources, such as participant data provided by pharmaceutical-industry sponsors, combined with reports from health authorities, real-world data, and information obtained from social media and the study participant data reflected in scientific publications, which is potentially linkable to the original identity of a specific individual, can elevate the risk of reidentification [12].

Gaining scientific insights requires the use of clinical trial data in a research context and disclosure of the data through scientific publications. However, this can create a risk that such data could be used for purposes beyond the scope of scientific research as several seemingly anonymous datasets were reported to be re-identified [19, 22], thereby jeopardizing the privacy of clinical trial participants and posing considerable ethical concerns, specifically in the case of re-identified genomic information, which could potentially trigger classification of some patients as being “less profitable or more expensive” [23]. Therefore, we determined a set of criteria based on a technology neutral, more conservative anonymization approach [12, 14] to identify risk and a process for minimizing reidentification risk to address such privacy concerns for clinical trial publications (manuscripts, abstracts, posters, presentations) prior to their availability in the public domain.

## Methods

### Scope of analyses

Takeda’s Clinical Trial Transparency (CTT) team developed an internal review process for analysing potential data reidentification risk in clinical trial materials containing information from interventional phase 1–4 clinical trials and destined for the public domain, such as abstracts, manuscripts, posters, and oral presentations. Takeda’s CTT review process applies a set of criteria allowing for the identification and minimization of data reidentification risk or privacy concerns. This review takes place prior to submission for publication in scientific peer-reviewed journals with open access, or presentation at medical conferences. A tracking tool was developed to cover 1) company publications that had been reviewed by CTT containing study participant information and 2) the outcome of the CTT review and analyses of these publications. The period chosen to collect data for the purpose of the present evaluation (1 January 2019 to 12 July 2021) reflects the period from introduction of the CTT process as a companywide standard until initial evaluation of information collected in the tracking tool approximately 1.5 years post-process initiation and tool implementation. Publications listed in the company’s tracking tool and which were within the scope of this analysis (N = 530), had the following characteristics: 1) were derived from clinical trials related to indications or diagnoses included on www.orpha.net or with a prevalence of diagnosis of fewer than 50 cases in 100 000 people (1:2000); or 2) included a sample size of treatment groups of fewer than 12 participants; or 3) included participant indirect identifiers or quasi‑identifiers in the text, tables or figures of the publication. These assessment criteria were also used to evaluate whether the data presented in the publications would be acceptable to be shared with a third party for further/secondary research purposes following an appropriate data request. The publications were reviewed from a data sharing and privacy perspective by clinical trials transparency subject matter experts prior to submission for publication in journals or presentation at conferences.

### Analyses of potential data reidentification risk

To protect study participant privacy, risk-based de-identification basic principles were used [24]. Publications were screened for direct identifiers (with the aim of deleting the direct identifiers or proceeding with pseudonymization) as well as for indirect identifiers or quasi-identifiers to assess the risk of reidentification, to quantify risk for data reidentification attacks (i.e., when background information is information obtained by a person/organization who intends to use it to attempt reidentification) targeting identification of personal information. The most conservative attack type relates to data already in the public domain, that is, when background information exists and someone may use this information to attempt reidentification of data [24]; for this type of attack an overall risk threshold of 0.09 was assumed [16] and also chosen for the purpose of the presented analyses as a conservative figure. K-anonymity is the most common criterion to protect against re-identification and is based on the equivalence class sizes of participants that have the same indirect identifier values, and hence cannot be distinguished from each other in the data to be published. The size of the equivalence class in the data set is at least k, and the re-identification probability will be at most 1/k [24, 25]. The larger the equivalent class size, the higher the protection of the study participants. If a risk threshold of *p* = 0.09 (1/11) is assumed, k needs to be 11. Therefore, a group of 11 participants with the same age, sex, race, geographic origin, and disease will have k = 11 and the probability of re-identification can be at most 1/11 (*p* = 0.09). For equivalent class sizes not described in the reviewed publication through tables or listings in combination with participant characteristics, the maximum risk for a data attack, according to the risk-based de-identification methodology [24] was estimated using overall assumptions for the given quasi-identifier. Based on the decision to declare treatment groups of fewer than 12 participants as a risk, we defined all studies that had less than 12 participants in their treatment groups or cohorts as having a risk-associated sample size. In our experience, for studies with a sample size of > 100, the likelihood for such small cohorts/groups with less than 12 participants is very low; therefore, we applied our estimates primarily to studies that had < 100 participants (“medium” sample size: 25–100 participants). In addition, we defined a sample size of < 25 participants per trial as being “small”, since in our reviews we saw a high likelihood of finding treatment or cohort groups with less than 12 participants in such studies.

With the restriction of two quasi-identifiers named concurrently for single individuals, equivalence class sizes were assumed to be higher than 11 on average, leading to a risk level within an acceptable range in relation to the risk threshold (*p* ≤ 0.09). To check compliance with the protection of privacy of participants in clinical trials, a specification checklist for review of scientific materials before submission for publication was developed based on standards [16, 17] adapted to select identifiers that are applicable to publication materials which, in brief, considered the following rules: elimination of all direct identifiers (participant ID, participant initials) and use of indirect identifiers or quasi-identifiers (e.g. sex, age, race, ethnicity, weight, body mass index [BMI]), avoidance of individual participant listings, use of parametric over non-parametric statistics and exclusion of geographical study locations. The full checklist is given in Additional file 1: Table S1 (Supplementary Information). Furthermore, clinical trial information in the public domain was searched using pre-defined search terms such as trial identifiers (company internal clinical trial ID numbers, ClinicalTrials.gov identifiers/ NCT number), study-specific terms (e.g. product ID/name/treatment, disease, patient population, indication) and criteria applicable to the data privacy of study participants (participant indirect identifiers or quasi-identifiers) according to the needs identified for each individual company manuscript reviewed. The internet search was focused on data and text that was publicly available including, e.g., demographic parameters, age, sex and ethnicity which were considered as increasing the risk of study participant identification [16, 17]. A clear understanding of the data environment (i.e., open access journal, versus in-person poster presentation) was reported as being an important factor in ensuring data protection, in addition to k-anonymity [15]. The above listed search terms were chosen to ensure that relevant information on previously published articles and scientific materials available in the open access online environment was not missed. The search was performed using the Google search engine and specific databases: PubMed, the regulatory clinical trial public registries ClinicalTrials.gov, EudraCT and the European Union Register of Post-Authorization Studies (EU PAS/ENCePP), and databases of the regulatory authorities the European Medicines Authority [EMA] and Health Canada. The information obtained from the search was compared with the data for individual participants in each company’s manuscript intended for publication and the potential impact on participant privacy was assessed.

In 2017, the International Committee of Medical Journal Editors issued a requirement for manuscripts submitted to its member journals reporting the results of clinical trial data to include a data sharing statement [26]. This was considered an ethical obligation because clinical trial participants put themselves at risk by participating in a clinical trial. Following this requirement, affected manuscripts must contain a data sharing statement reflecting the availability of the data for further/secondary research purposes. To meet this standard, a data sharing statement was to be included in all relevant public materials.

Although the clinical trial data are to be provided for third-party research after de-identification, in compliance with applicable privacy laws, data protection and requirements for consent and anonymization, there is a reasonable likelihood that study participants could be reidentified in certain instances. The data sharing statements included in the reviewed publications were therefore assessed from a privacy perspective (using predefined criteria) and adjusted according to the risk level for reidentification.

## Results

### Scope of analyses

In total, 530 publications were reviewed from 1 January 2019 to 12 July 2021. The number of publications reviewed by our team has increased rapidly since the process for analysing potential data reidentification risk was introduced in the company in 2019. Most of the publications assessed for reidentification risks were manuscripts (N = 275) followed by abstracts (N = 110), posters (N = 87), and oral presentations (N = 58) (Table 1). Therefore, more than 50% of the publications assessed were manuscripts.

**Table 1:** Type and number of publications reviewed for analysis of potential data reidentification risk

Type of publication	2019	2020	2021 (1 January–12 July)	Total
Manuscript	75	116	84	275
Abstract	26	43	41	110
Poster	38	40	9	87
Presentation	4	20	34	58
Total	138	219	168	530

### Outcome of data reidentification risk analyses

**Fig. 1:**
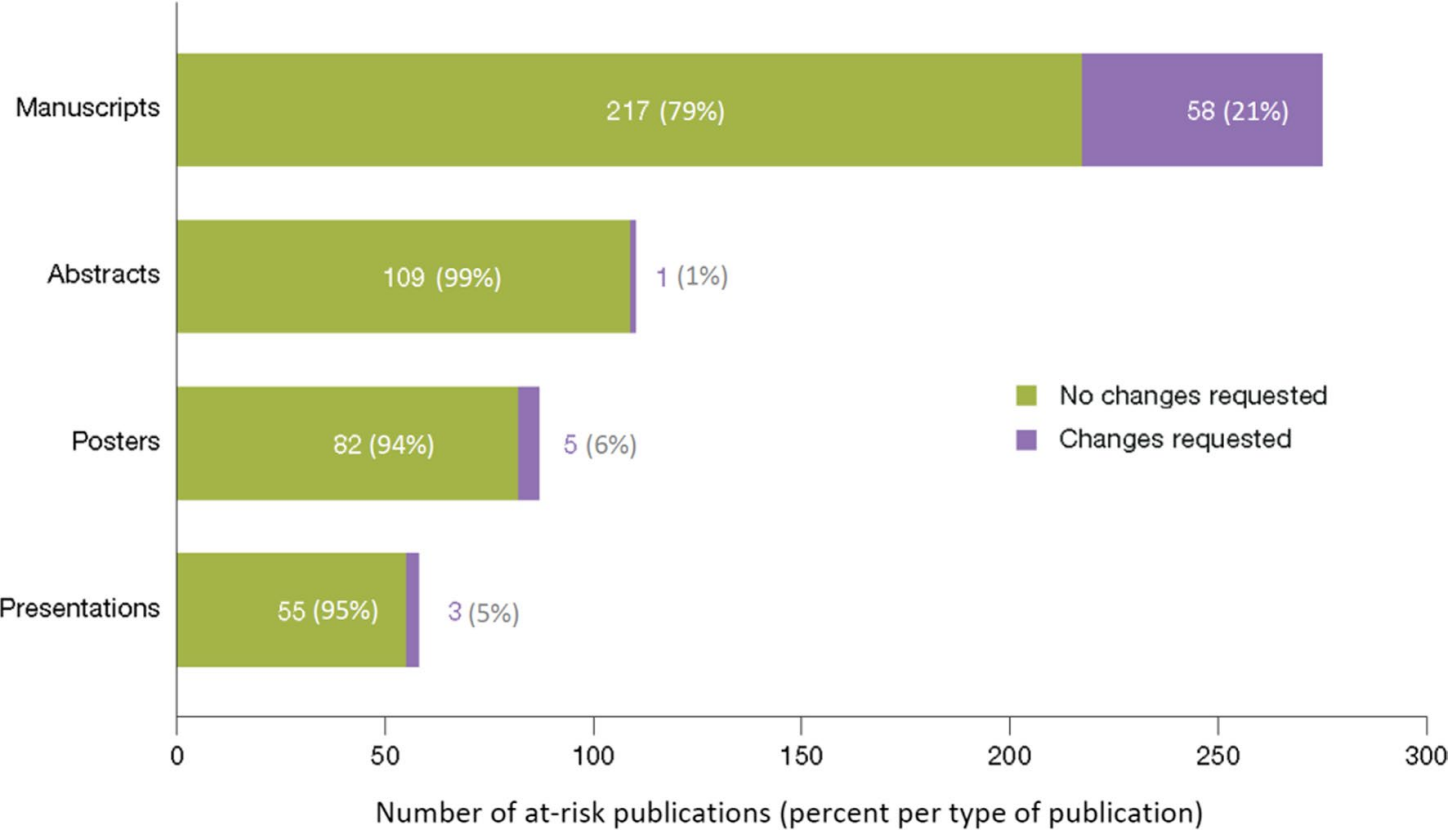
Findings from a review to identify publications at risk for study participant reidentification

In total, 87% (463/530) of publications reviewed between January 2019 and July 2021 required no changes to minimize the risk of reidentification of individuals. Using the specification checklist for review of scientific materials and the data sharing statement criteria, a relatively low percentage of the publications assessed were found to require changes (13% [67/530]; Fig. 1). Most of these changes affected manuscripts rather than conference materials: 58 (21%) of the 275 manuscripts reviewed were affected; only 5 (6%) of the 87 posters and 3 (5%) of the 58 presentations required changes.

**Fig. 2:**
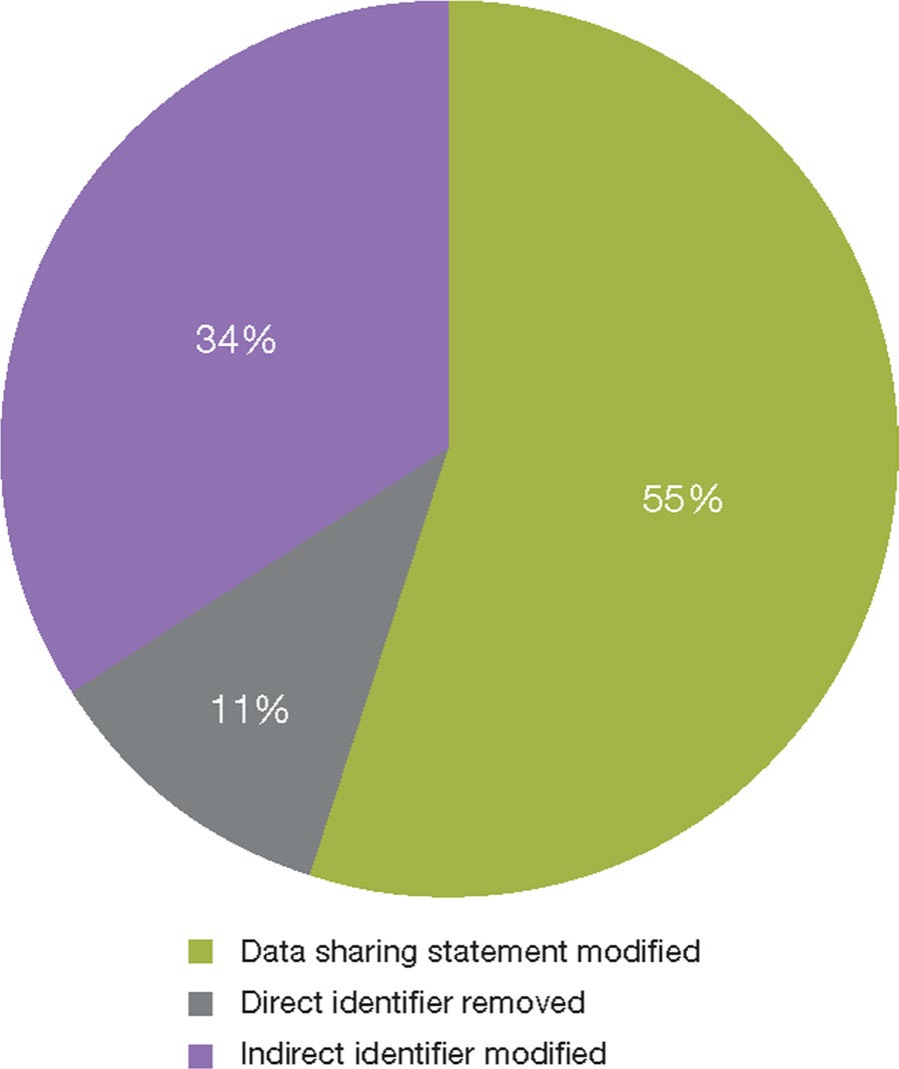
Types of changes introduced in publications when changes were required. 76 changes were required in 67/530 publications to minimize the risk of reidentification of study participants (direct identifier removed, n = 8; indirect identifier modified, n = 26; data sharing statement modified, n = 42)

As shown in Fig. 2, the most prominent findings on review related to data sharing statements (e.g., the data sharing statement did not comply with the predefined standards owing to the lack of an anonymization statement; improper timing for data availability; inappropriate interpretation of requirements for consent), which were modified to ensure protection of privacy for study participants and to present a consistent message in the public domain. Data sharing restrictions were applied if the publications included data for patients with rare diseases or highly sensitive data (e.g., genetic data, illicit drug use, neuropsychiatric disorders) or if pre-specified interim results were presented.

Approximately one-third, 34%, of the implemented changes (26/76) concerned the use of indirect identifiers. Direct identifiers comprised 11% of the implemented changes (8/76); these were the most serious findings because they could jeopardize the privacy of the study participants. As the pattern of recognition resulting from the use of direct and indirect identifiers, these two types of findings are described in detail below.

Direct identifiers were found in eight manuscripts. Participant IDs were identified in text (n = 3), figures (n = 3) and tables (n = 2) of manuscripts (Table 2). When participant IDs were discovered, the implemented changes were mainly to transform them by renumbering consecutively or replacing with random letters.

**Table 2:** Direct identifiers detected in manuscripts and changes to minimize risk of reidentification of study participants

Location in manuscript of direct identifier (incidence)	Cause/trigger	Implemented change
Body text (three manuscripts)	Several direct identifiers in text proposed Single ‘participant IDs’ used in text and footnotes of tables Term ‘participant ID’ used for consecutive number	Inclusion of direct identifiers in text rejected Transformed ‘participant IDs’ into consecutive numbers ‘Participant ID’ label changed to participant number to avoid misleading interpretations
Figures/listings (three manuscripts)	‘Participant IDs’ in figures (in two manuscripts) ‘Participant IDs’ in figures; use of genotype information in participant listings	Transformed ‘participant IDs’ into consecutive numbers Transformed ‘participant IDs’ into consecutive numbers and removed genotype table
Tables (two manuscripts)	‘Participant IDs’ in table	Transformed ‘participant IDs’ into consecutive numbers

Indirect identifiers were found in 26 publications. Two categories of indirect identifiers required the most changes in the reviewed publications: personal characteristics and location/geographical characteristics (Table 3). Although the use of a single indirect personal identifier (e.g., sex, age, race, weight, BMI) would not allow study participant reidentification per se, if several of these identifiers are presented together with participant-specific data, there is an increased risk of participant reidentification. The risk exposure of study participants correlates with the sample size. Therefore, for studies with small to medium sample sizes, and treatment cohorts/groups of fewer than 12 participants, no more than two quasi-identifiers (e.g., sex and age) were permitted. The recommended risk level of 0.09 allowed for the use of maximum two identifiers in order to achieve k-anonymity and remain consistent with the conclusions of the EU Data Protection Working Party [12], also minimizing the linkability associated with individual data re-identification. If the location/country in which the study was conducted was provided, then the number of individual identifiers was further reduced to one, either sex or age. Descriptions of characteristics in aggregated data and summary statistics were generally not considered indicators for reidentification. However, exceptions were made for aggregated data presented in cross-tabulations with multiple categories (e.g., three age groups in four treatment groups). In some publications, the small number of cases meant that the total number of participants under certain demographic characteristics (e.g., study locations, country, or state) was only one or two. Such gaps were primarily identified in tables presented in manuscripts. To resolve this issue, a table split was applied in which demographic characteristics were presented only for the total sample and the disease characteristics (which no longer contained indirect personal identifiers) were broken down into multiple categories. The lack of striking deviations across treatment groups in demography was addressed in the body text of the manuscript.

**Table 3:** Indirect identifiers detected in publications and changes to minimize risk of reidentification of study participants

Category of indirect identifier (incidence)	Cause/trigger	Implemented change
Personal participant characteristics (19 publications)	Age, gender, race, age at diagnosis and BMI listed in demographic information	Anonymized demographic listing by modifying specific age information
	Report of early gene therapy data on only two study participants	Removed individual data for two cases from an ongoing study
	Age, gender, race, ethnicity, weight, and BMI cross-tabulated by treatment groups resulting in multiple n = 1 and n = 3 cell frequencies*	Split demographic table to show identifier only in an overall frequency and list only disease characteristics across treatment groups
	Age, gender, race, and country of origin in one single participant listing	Removed race and country of origin and left only age and gender
	Age in description of individual participants	Removed exact age from individual participant description
	Cell sizes for gender, age and race were small owing to a detailed age group cross‑tabulation	Removed race from the demographics table to reduce indirect identifiers to sex, age, and US origin
	Age, sex, and race included in the comparison and descriptions of different treatment conditions and outcome findings	Removed race to avoid exposure of specific ethnicity
	Nationality of two participants in country with low enrolment	Removed nationality in text for the description of protocol deviations
	Age, gender, race, ethnicity, weight, and BMI cross-tabulated in a multiple-treatment group causing very small cell sizes	Presented demographic identifiers only for the overall group and made cross‑tabulations for the remaining disease parameters (table split)
	Age, gender, race, and BMI cross-tabulated across multiple treatment groups causing n = 1 frequency	Presented demographic identifiers for the overall group and made cross‑tabulation from the remaining disease parameters (table split)
	Detailed information on family relationships and ethnic background of participants and original participant identifiers (such as age, gender) used	Removed details regarding family relationships and ethnic background (showed only indicator ‘yes’ for existing family history), participant identifiers replaced with generic identifiers (e.g., participant numbers)
	Individual participant descriptions: age and sex in study with small group sizes	Removed age from the individual participant descriptions and left only gender information
	Information related to time, location and investigator contained in computed tomography scans and liver biopsy images	Removed all indicators related to time, location and investigators from scans and images
	Age, gender, race, and ethnicity listed in demographics table causing multiple small cell sizes in the table	Removed gender as identifier from the table
	Age, gender, and country list for each participant	Age was recoded to age groups
	Age, gender, and date of death listed in one table with therapy received and cause of death	Removed exact date of death
	Exact dates (treatment, SAEs, and diagnosis) in several listings	Removed all dates, but kept study day information
	Age and gender listed with each arterial occlusive event per participant	Removed gender information from the listing of arterial occlusive events
	Three indirect identifiers used to describe a single participant	Removed age or gender
Location/geographical characteristics (seven publications)	Site numbers (containing part of the participant IDs) reported	Removed site numbers from manuscript as they are usually part of the participant identification number
	Investigator names, sites, country, and frequency of participants enrolled	Removed number of enrolled participants per site and left only list of countries
	Investigator names (four publications)	Investigator names removed
	Site number as part of the participant number	Removed site numbers by transforming to consecutive numbers

*BMI* Body mass index, *SAE* Serious adverse event

^*^The smaller the sub-group size (cell size) the higher the risk of re-identification (exposure risk)

In addition, an Internet search protocol was performed to establish whether a combination of the data and information included in the manuscript (e.g. demographic parameters, age, sex and ethnicity), together with data provided in publicly available regulatory documents (CSRs, Module 2 clinical overview/summaries of the CTD within the scope of EMA transparency regulations [10], public assessment reports within the scope of the Regulation (EC) No 1049/2001)) [27] would increase the risk of study participant identification. Our search showed that in several manuscripts and posters, the additional data identified in the public domain were concerning, as targeted mapping of direct and indirect identifiers could enhance the individual participants’ profiles; changes were therefore required to these publications to ensure protection of the privacy and anonymity of the clinical trial participants.

## Discussion

Initiatives aimed at increasing transparency and data sharing of pharmaceutical-industry-sponsored clinical research are promoting scientific collaboration and strengthening public trust. However, large-scale collection and publication of detailed study participant-level clinical data raises legitimate privacy concerns. To date, little is known about the availability of internal pharmaceutical industry processes and standards to review clinical materials prior to submission for publication in journals or presentation at medical conferences. To the best of our knowledge, Takeda is a pioneer among biopharmaceutical companies in having developed a review process for clinical materials destined for the public domain to prevent data privacy issues. In this paper, we describe a process for analysing and minimizing the risk of potential study participant reidentification in scientific publications that report clinical trial data. Our analysis focused on the detection of individual identifiers that may allow participant reidentification, as well as the evaluation of data sharing statements describing data availability for secondary research. A relatively low percentage of the publications reviewed (13%) were found to require changes. Most changes were to data in manuscripts (21% of the manuscripts reviewed required changes), which is not surprising, considering that a greater sum of textual information, tables and figures containing potentially identifiable data in these publication types exists, and therefore increases the likelihood of data reidentification. Changes were required less often in abstracts, posters, and presentations: 0.9%, 5.8% and 5.2% of the reviewed abstracts, posters, and presentations, respectively, required changes. This may be partly due to the more detailed nature of the data presented in manuscripts than that in abstracts, posters, and oral presentations, which contain mainly non-identifiable aggregated data and/or summary statistics.

It is known that the risk of participant reidentification increases with the number of available indirect identifiers [28]. Therefore, our analysis focused on reducing the number of indirect identifiers in publications, especially for study samples that were small to medium in size. Description of participant characteristics using aggregated data and summary statistics were not considered indicators or risks for participant reidentification, but in our approach some additional measures were introduced for cross-tabulations with multiple categories (e.g., different identifiers in different sources), when the combination of raw data could allow identification for some study participants. Special attention was also given to the detection of participant IDs in the publications. To eliminate risk of reidentification, these IDs were converted to a proxy ID whenever detected (i.e., they were renumbered using a method such that the key code connecting a person/individual and a study participant was broken, ensuring that a participant’s identity could not be reconstructed).

Our analysis also assessed the potential for study participant data in publications to be matched or linked to other relevant data available in the public domain. Under EMA and Health Canada transparency policies, clinical trial reports and summaries (Module 2—clinical section of a CTD) that are part of regulatory applications and are assessed in support of marketing authorizations are subject to publication [16, 29]. These clinical reports typically include individual study participant data quasi-identifiers, such as dates of admissions, geographical location, demographic information, socioeconomic information, rare diagnoses, concomitant medications and diseases, adverse events/reactions, and dates of hospitalization or death. Similarly, such anonymized variables are important parameters commonly included in scientific publications. Although these data are anonymized, they can become problematic if data mapping and triangulation are applied [27, 30, 31]. In several instances, the internet searches described as part of our analyses revealed an increased risk of study participant identification when data and information presented in manuscripts (e.g. demographic parameters, age, gender and ethnicity) were combined with data provided in publicly available clinical regulatory documents (CSRs, Module 2 clinical overviews/summaries of CTD within the scope of EMA transparency regulations [16] and public assessment reports within the scope of EU policy on access to documents [32]). These findings clearly demonstrate why regulatory bodies and data protection agencies [12] focus on both the data and the environment in which it is released to ascertain realistic measures of risk [15]. The sufficiency of anonymization of study participants data presented in scientific articles depends not only on the properties of the data themselves but also on its open, uncontrolled environment when it is published in open access and becomes widely available on the Internet [15]. Although only anonymized and de-identified clinical trial data were used in both the scientific publications and the publicly available clinical regulatory documents (which were processed up to several levels with data redaction, anonymization, and removal), when these sources were matched to each other there was an increased risk that individuals could be reidentified and/or the data could be used for purposes beyond the scope of scientific research. Our analyses demonstrated that the inclusion of combined direct and indirect identifiers provided an extended study participant profile, thus jeopardizing study participant privacy. If study participants’ data released in the public domain through scientific publications is not sufficiently secured, then harm to individuals, a break in participants’ trust, and damage to the research process itself could result [33]. Academic medical journals are increasingly requiring that authors give the research community access to anonymous data used in published articles, as well as access to related clinical trial information. This data sharing increases the pool of openly accessible and connected/mapped clinical trial data and will likely lead to study participant data appearing in secondary/tertiary publications. However, in the event that the risk of reidentification becomes intolerable, data sharing restrictions should be considered, and hence our analysis also included criteria that could be used to determine data availability for further research purposes. Of note, more than half of the data privacy findings detected in this analysis were related to data sharing statements. These data sharing statements often presented either information consistency issues or privacy risks for the study participants, and adjustments were required to safeguard sensitive data, particularly for patients with rare diseases.

## Conclusions

Given the rapid increase in recent years in the amount of publicly accessible clinical trial data, and the introduction of various regulatory policies concerning such data across the globe (for example, EMA transparency policy on the publication of clinical data; EU clinical trial regulations; and Health Canada’s regulation on the public release of certain clinical trial information), the need to ensure the privacy of study participants has become an important focus. In this respect, publications need to be systematically evaluated: clinical trial participant data must be subject to risk minimization and sharing of such data must be carefully controlled. Pharmaceutical companies could consider our approach and benefit from implementing a process for risk assessment prior to publication, especially for materials focused on clinical trials in sensitive rare disease populations to be made available in the public domain. The use of analyses, such as the one described here, would be a valuable starting point to prevent reidentification of study participants from the clinical data reported in scientific publications, thereby ensuring that the rights of individuals with regard to their personal data are sufficiently protected. Risk-minimization strategies, such as the de-identification of clinical trial data in scientific publications and the use of controlled data sharing conditions, can promote protection of privacy for study participants.

## Supplementary Information

**Additional file 1.** Specification checklist for review of scientific materials before submission for publication.

## Data Availability

The authors confirm that all relevant data are included in the article.

## Abbreviations

BMI: Body mass index
CSR: Clinical study report
CTD: Common technical document
EMA: European Medicines Agency
ENCePP: European network of centres for pharmacoepidemiology and pharmacovigilance
EU: European Union
EudraCT: The European Union drug regulating authorities clinical trials database
