# Supplementary material for: Data privacy protection in scientific publications: process implementation at a pharmaceutical company
Source: BMC Med Ethics. 2022 Jun 25;23:65. doi: 10.1186/s12910-022-00804-w (PMC9233846; doi:10.1186/s12910-022-00804-w)
Supplement: Supplementary file 1 — Additional file 1. Specification checklist for review of scientific materials before submission for publication. [file 12910_2022_804_MOESM1_ESM.docx]

**Table S1** Specification checklist for review of scientific materials before submission for publication

| **Rule number** | **Rules designed to ensure compliance with protection of privacy for participants in clinical trials** |
| --- | --- |
|  | Eliminate all direct identifiers (participant ID, initials of participant) and replace with dummy numbers or random letters. Participant IDs provide direct protection of the clinical study participants but because there is pseudo-randomization (usually only a sequential assignment) with a permanent and known 1:1 relationship between the person and participant in place, the participant ID was seen as a direct personal identifier as it relates to data sharing activities [27] |
|  | Use indirect identifiers or quasi-identifiers (sex, gender, age, race, ethnicity, weight, BMI etc.) to characterize study samples only with descriptive statistical measures that represent groups of individuals |
|  | Avoid individual participant listings with a standard set of indirect identifiers (e.g., sex, age, race, weight, BMI). If such a listing is indispensable, a maximum of two identifiers only (e.g., sex and age) can be used to describe study participants in addition to their disease characteristics |
|  | Participant characteristics description tables to be used for main treatment groups only, while avoiding further cross-tabulation into very small sized subgroups |
|  | Parametric statistics (mean, standard deviation) are preferable compared with non‑parametric measures (median, quarter 2–quarter 3, minimum–maximum), depending on distribution, and: |
|  | A total of no more than three participants per table cell for any given characteristic is to be avoided for non-parametric measures in such tables |
|  | A total of one participant per table cell for any given characteristic is to be avoided for parametric measures in such tables |
|  | Geographical study locations (e.g., country or state/province), if included in the publication, are to be treated as an additional indirect identifier |
